# Conversions between metabolically unhealthy and healthy obesity from midlife to late-life

**DOI:** 10.1038/s41366-023-01425-y

**Published:** 2023-12-02

**Authors:** Peggy Ler, Elsa Ojalehto, Yiqiang Zhan, Deborah Finkel, Anna K. Dahl Aslan, Ida K. Karlsson

**Affiliations:** 1 Department of Medical Epidemiology and Biostatistics, Karolinska Institutet, 17177 Stockholm, Sweden; 2 School of Public Health (Shenzhen), Sun Yat-Sen University, Shenzhen, China; 3 Center for Economic and Social Research, University of Southern California, Los Angeles, CA USA; 4 School of Health and Welfare, Box 1026, Jönkoping University, 55318 Jönköping, Sweden; 5 School of Health Sciences, University of Skövde, 54128 Skövde, Sweden

**Keywords:** Metabolic syndrome, Obesity

## Abstract

**Introduction:**

Metabolically healthy obesity may be a transient phenotype, but studies with long follow-up, especially covering late-life, are lacking. We describe conversions between cross-categories of body mass index (BMI) and metabolic health in 786 Swedish twins with up to 27 years of follow-up, from midlife to late-life.

**Methods:**

Metabolic health was defined as the absence of metabolic syndrome (MetS). We first visualized conversions between BMI-metabolic health phenotypes in 100 individuals with measurements available at ages 50–64, 65–79, and ≥80. Next, we modeled conversion in metabolic health status by BMI category in the full sample using Cox proportional hazards regression.

**Results:**

The proportion of individuals with MetS and with overweight or obesity increased with age. However, one-fifth maintained a metabolically healthy overweight or obesity across all three age categories. Among those metabolically healthy at baseline, 59% converted to MetS during follow-up. Conversions occurred 56% more often among individuals with metabolically healthy obesity, but not overweight, compared to normal weight. Among those with MetS at baseline, 60% regained metabolic health during follow-up, with no difference between BMI categories.

**Conclusions:**

Conversions between metabolically healthy and unhealthy status occurred in both directions in all BMI categories. While conversions to MetS were more common among individuals with obesity, many individuals maintained or regained metabolic health during follow-up.

## Introduction

Metabolically healthy obesity (MHO) refers to a body mass index (BMI) above 30, but preserved metabolic health. There are no universal criteria to identify MHO, but metabolic health is often defined as the absence of metabolic syndrome (MetS) [1]. Although the risk of type 2 diabetes (T2D) and cardiovascular disease may be higher in MHO than in metabolically healthy normal weight, it is considerably lower than for metabolically unhealthy obesity [1]. However, MHO is considered a transient state, where individuals often develop MetS over time, followed by an increased risk of cardiovascular disease and T2D [2]. Few studies have examined conversions between BMI and metabolic health phenotypes over long time periods, and most cohorts have comparatively young ages at baseline [2]. As metabolism undergoes substantial changes during aging [3, 4] and BMI tend to decline at higher ages [5], it is not clear whether the stability or transiency of MHO in late-life is comparable to that of younger age groups. Therefore, we used longitudinal data from older adults with up to 27 years of follow-up, and aimed to: (1) describe conversions between cross-categories of BMI and metabolic health (BMI-MH) phenotypes from midlife (age 50–64), to early late-life (age 65–79), and late late-life (age ≥80), and (2) examine if conversions from metabolic health to MetS, or from MetS to metabolic health, are more common in some BMI categories.

## Material and methods

The study population comprises 859 individuals from the Swedish Adoption/Twin Study of Aging (SATSA), a cohort study of same-sex twin pairs over the age of 50 who participated in up to 10 waves of data collections between 1986 and 2014 [6]. Each wave included an extensive questionnaire, interview, health examination, and blood sample collection, from where self-reported disease diagnoses and medication use and measures of height, weight, blood pressure, and blood biomarkers were available [6, 7]. Participants provided informed consent, and the study was approved by the Regional Ethical Review Board in Stockholm.

BMI was categorized (based on measured height and weight as kg/m^2^) into normal weight (18.5–24.9), overweight (25–29.9), and obesity (≥ 30). We used measured blood pressure, blood biomarkers, and self-reported disease diagnoses and medication use to define metabolic health as having ≤1 of the following (based on the NCEP ATP-III criteria [8] for MetS): hypertension (systolic or diastolic blood pressure ≥135 or ≥85 mmHg, respectively), hyperglycemia (fasting blood glucose levels ≥6.1 mmol/L, self-reported diagnosis of T2D, or self-reported use of T2D medications), hypertriglyceridemia (fasting triglyceride levels ≥1.70 mmol/L or self-reported use of lipid-lowering medications), and low high-density lipoprotein cholesterol (HDL-C levels <1.03 mmol/L in males and <1.30 in females, or self-reported use of lipid-lowering medications). Individuals were grouped into cross-categories of BMI-MH phenotypes: metabolically healthy normal weight (MHNw), overweight (MHOw), or obesity (MHOb), or metabolically unhealthy normal weight (MUNw), overweight (MUOw), or obesity (MUOb). To describe conversions between BMI-MH phenotypes across aging, we determined BMI-MH status in three age groups: 50–64, 65–79, and ≥80 years. When more than one measure was available in the same age group, that closest to age 57, 72, and 87 was selected for the respective age group. One hundred individuals had measures in all three age groups, and their conversions between BMI-MH phenotype across aging were visualized with the ggalluvial package [9] in R 4.0.5 [10]. To examine differences in conversion rates between metabolically healthy status and MetS by BMI category, we included 739 individuals with at least two measurements available. We modeled baseline BMI category as exposure and change in metabolic health status as the outcome in Cox proportional hazard regression with age as the underlying timescale, using STATA 17.0 [11]. Individuals were followed from the first measurement occasion to the first occasion when their metabolic health status differed from that at baseline, or to death or last participation (whichever occurred first). An interaction term was included to stratify by baseline metabolic health status so that the rate of conversion in metabolic health status was compared to those with the same metabolic health status and normal weight at baseline. To account for major confounders, all models were adjusted for sex, education (≤ 7 years versus >7 years, corresponding to basic versus more than basic education at the time), and smoking (never versus ever smoker). Robust standard errors were applied to account for non-independent observations (relatedness between twins).

## Results

### Description of conversions between BMI-MH categories

**Fig. 1:**
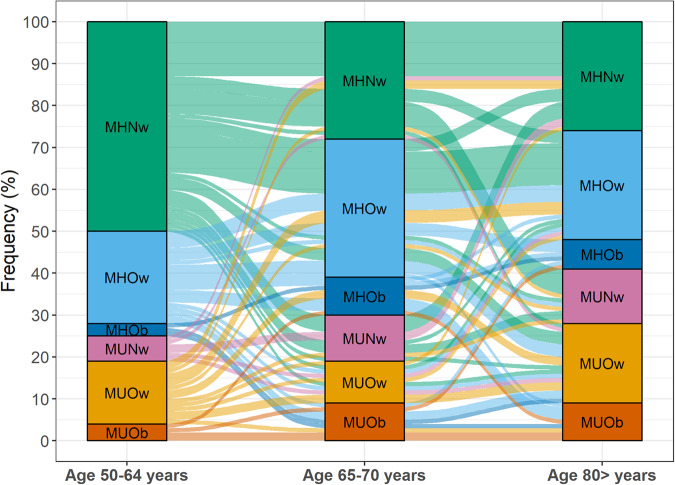
Conversions between BMI and metabolic health phenotypes from midlife through late-life. One hundred individuals with measurements in each age category were included. The height of the boxes and thickness of the paths are proportional to the number of individuals in each category. Paths are colored by baseline category. MH metabolically healthy, MU metabolically unhealthy, Nw normal weight, Ow overweight, Ob obesity.

Figure 1 presents BMI-MH phenotype across time for the 100 individuals with measures taken in all three age categories, demonstrating that conversions are common in all directions across aging. The proportion of metabolically unhealthy individuals increased over time, as did the proportion of individuals with overweight or obesity. Twenty-eight percent of individuals with MHNw at baseline remained MHNw throughout the follow-up. Eighteen percent of those with MHOw at baseline remained MHOw across follow-up, and one of the three individuals with MHOb at baseline remained MHOb. In total, 30%, 50%, and 67% of those with MHNw, MHOw, and MHOb at age 50–64 had converted to MetS by age ≥80, respectively. On the other hand, of those with MUNw, MUOw, and MUOb at age 50–64, 67%, 47%, and 25%, respectively, had converted to being metabolically *healthy* by age ≥80.

### Conversion between metabolically healthy status and MetS by BMI category

At first participation, the 739 individuals with at least two measurement occasions had a mean age of 63.3 (standard deviation (SD) 8.3, range 50–88); a mean BMI of 25.7 (SD 3.8, range 17.3–46.1); 32.9.% had MetS; 59.1% were women; 53.0% had less than 7 years of education; and 50.9% were ever-smokers. The mean follow-up time was 14.7 years (SD 7.6, range 2.9–27.0).

Table 1 shows BMI-MH phenotype at baseline, and metabolic health status at the end of follow-up. Conversions of metabolic health status occurred in both directions and all BMI categories. Among the 490 individuals who were metabolically healthy at baseline, 283 (58%) converted to MetS during follow-up. Compared to those with MHNw at baseline, conversions to MetS occurred at 56% higher rate among those with MHOb, but not MHOw, at baseline (Table 1). Among the 242 individuals with MetS at baseline, 146 (60%) regained metabolic health during follow-up, with no statistically significant difference between BMI groups (Table 1).

**Table 1:** Conversions between metabolic health and MetS by baseline body mass index and metabolic health status.

Baseline		End of follow-up		Difference in rates of conversion
Phenotype	*N*	Healthy, *N* (%)	MetS, *N* (%)	HRR (95% CI)
Metabolically healthy				
Normal weight	272	122 (45)	150 (55)	1 (ref)
Overweight	176	74 (42)	102 (58)	1.01 (0.78–1.30)
Obesity	42	11 (26)	31 (74)	1.56 (1.02–2.37)
Metabolically unhealthy				
Normal weight	70	46 (66)	24 (34)	1 (ref)
Overweight	126	75 (60)	51 (40)	0.90 (0.63–1.28)
Obesity	46	25 (54)	21 (46)	1.29 (0.79–2.13)

Number of individuals in each body mass index category by metabolic health status at baseline, at the end of follow-up, and the hazard rate ratio (HRR) and 95% confidence intervals (CI) of conversion of metabolic health status during follow-up (from metabolically healthy at baseline to metabolic syndrome (MetS) during follow-up, or from MetS at baseline to metabolically healthy during follow-up). HRRs were obtained from Cox proportional hazards regression with BMI category as predictor and conversion in metabolic health status as the outcome. An interaction term was included to stratify by baseline metabolic health status. Age was used as the underlying timescale, and the model additionally adjusted for sex, education, smoking, and relatedness among twins.

## Discussion

In a sample of older adults with up to 27 years of follow-up, we first qualitatively described conversions between BMI-MH phenotypes from midlife (age 50–64) to early late-life (age 65–79) and then to late late-life (age 80 and above). In line with previous studies of adult samples [2, 12, 13], many individuals with MHOw or MHOb converted to MetS over time, but around one-fifth remained metabolically healthy from midlife and through early late-life and late late-life. Secondly, we tested for differences in the occurrence of conversions in metabolic health status between the different BMI categories. More than half of those who were metabolically healthy at baseline converted to MetS during the follow-up in all BMI categories. Individuals with MHOb at baseline were more likely to convert to MetS compared to those with MHNw at baseline, while no difference was seen between those with MHNw and MHOw. Interestingly, conversions from MetS to metabolically healthy BMI categories were also common, with no statistically significant difference between BMI categories.

The aging process is linked to major physiological changes, including changes in metabolism [3, 4] and BMI [5]. While transitions between BMI-MH phenotypes have been examined previously, most studies have focused on the stability of MHO in samples with a relatively young age at baseline [2]. Two previous studies have specifically modeled transitions between the six BMI-MH phenotypes, one followed 3512 women aged 50–79 over an average of six years [12] and the other 9742 men and women aged 20–60 over an average of 4 years [13]. In both studies, individuals with overweight or obesity were more likely to convert from metabolic health to MetS and less likely to convert from MetS to metabolic health, compared to those with normal weight. In the current study, we leveraged data from a well-characterized cohort with long follow-up, covering midlife to late late-life, to study conversions between BMI-MH phenotypes in older individuals over substantially longer periods than previously examined.

However, the small sample size limited our opportunities to examine the causes and consequences of conversions in metabolic health status or to apply more advanced models of transitions between BMI-MH phenotypes. It should also be mentioned that no consensus exists for how metabolic health should be defined [14]. While we used the NCEP ATP-III criteria [8] for MetS, the definition means that small changes in one of the constituents over time may lead to artificial changes in metabolic health status. Objective measures of BMI and metabolic health is a strength of the current study, but we also used self-reported information on diagnoses and the use of medications to define metabolic health, which may reduce the validity. In addition, the study sample consists of Swedish twins aged 50 or above, with a comparatively low proportion of MetS and obesity at baseline. As in most countries, the prevalence of obesity has risen substantially in Sweden during the past decades, from 9% in 1995 to 17% in 2017 [15], potentially limiting the generalizability of our findings to older adults today.

In conclusion, conversions between metabolic health and MetS were relatively common in both directions across all BMI categories during aging. Importantly, a substantial proportion of the sample maintained MHOw and MHOb throughout aging, and, in addition, a considerable proportion regained a metabolically healthy status. Taken together, this indicates that conversions between BMI-MH phenotypes seen in younger samples and over shorter follow-up periods [12, 13] continue across late-life. A better understanding of what predicts these conversions and the ability to maintain metabolic health in overweight and obesity could substantially improve obesity care and help promote and maintain metabolic health throughout life.
